# *Escherichia coli* NemA Is an Efficient Chromate Reductase That Can Be Biologically Immobilized to Provide a Cell Free System for Remediation of Hexavalent Chromium

**DOI:** 10.1371/journal.pone.0059200

**Published:** 2013-03-13

**Authors:** Katherine J. Robins, David O. Hooks, Bernd H. A. Rehm, David F. Ackerley

**Affiliations:** 1 School of Biological Sciences, Victoria University of Wellington, Kelburn Parade, Wellington, New Zealand; 2 Institute of Fundamental Sciences, Massey University, Tennent Drive, Palmerston North, New Zealand; 3 MacDiarmid Institute for Advanced Materials and Nanotechnology, Victoria University of Wellington, Kelburn Parade, Wellington, New Zealand; 4 Centre for Biodiscovery, Victoria University of Wellington, Kelburn Parade, Wellington, New Zealand; Instituto Nacional de Cardiologia, Mexico

## Abstract

Hexavalent chromium is a serious and widespread environmental pollutant. Although many bacteria have been identified that can transform highly water-soluble and toxic Cr(VI) to insoluble and relatively non-toxic Cr(III), bacterial bioremediation of Cr(VI) pollution is limited by a number of issues, in particular chromium toxicity to the remediating cells. To address this we sought to develop an immobilized enzymatic system for Cr(VI) remediation. To identify novel Cr(VI) reductase enzymes we first screened cell extracts from an *Escherichia coli* library of soluble oxidoreductases derived from a range of bacteria, but found that a number of these enzymes can reduce Cr(VI) indirectly, via redox intermediates present in the crude extracts. Instead, activity assays for 15 candidate enzymes purified as His6-tagged proteins identified *E. coli* NemA as a highly efficient Cr(VI) reductase (*k_cat_/K_M_*  = 1.1×10^5^ M^−1^s^−1^ with NADH as cofactor). Fusion of *nemA* to the polyhydroxyalkanoate synthase gene *phaC* from *Ralstonia eutropha* enabled high-level biosynthesis of functionalized polyhydroxyalkanoate granules displaying stable and active NemA on their surface. When these granules were combined with either *Bacillus subtilis* glucose dehydrogenase or *Candida boidinii* formate dehydrogenase as a cofactor regenerating partner, high levels of chromate transformation were observed with only low initial concentrations of expensive NADH cofactor being required, the overall reaction being powered by consumption of the cheap sacrificial substrates glucose or formic acid, respectively. This system therefore offers promise as an economic solution for *ex situ* Cr(VI) remediation.

## Introduction

Hexavalent chromium is generated as a water-soluble waste product by numerous industrial processes, including pigment production, leather tanning, wood preservation, and stainless steel manufacture. It is also a by-product of nuclear weapons manufacture, and at US Department of Energy waste sites is the second most abundant heavy metal contaminant [1]. Without human intervention, Cr(VI) has been projected to persist at dangerous levels at such waste sites for well over 1000 years [2]. Although Cr(VI) does not cause direct damage to DNA it is nonetheless a dangerous carcinogen due to its ability to penetrate cells via sulfate transporters, whereupon it is reductively activated to a variety of mutagenic and genotoxic intermediates [3]. In contrast, most cells are impermeable to Cr(III), which is generally insoluble under standard environmental conditions [4] and 1,000-fold less mutagenic than Cr(VI) in the Ames test [5].

A wide range of bacteria have been isolated that can reduce Cr(VI) to Cr(III) [6], offering promise for bioremediation as a cost-effective and environmentally friendly means to detoxify environmental Cr(VI) pollution. Bacterial Cr(VI) reduction can be both enzymatic and non-enzymatic, but either pathway is thought to unavoidably generate redox-active intermediates that inflict cellular damage and impact the viability of the remediating cell [7]–[10]. Some of the mechanisms that bacteria employ to defend themselves against Cr(VI) cytotoxicity, such as efflux or diminished Cr(VI) uptake [11], [12], are counter-productive to bioremediation. Furthermore, many contaminated sites are nutrient poor [13] and co-contaminated with multiple pollutants likely to inhibit bacterial growth [1]. Biostimulation by addition of long-lived electron donating compounds such as polylactates [14] is an attractive strategy to promote bacterial growth and Cr(VI) transformation, but runs the risk of generating large amounts of biomass that can clog subsurface pores and confine bioremediation to a narrow zone [15].

As an alternative remediation strategy we have sought to develop an enzyme-based system for use in detoxifying Cr(VI) contaminated groundwater *ex situ*. An effective cell free system for Cr(VI) transformation would sidestep the issues of Cr(VI) and co-contaminant cytotoxicity, and nutrient limitation, that restrict bacterial bioremediation. However, enzymes outside of the cellular environment are potentially subject to stability issues. As they also do not self-replicate and multiply like bacteria, a prerequisite for a viable enzymatic remediation strategy is that the enzyme be able to be prepared cheaply and in large quantities. Finally, it is essential that the enzyme be able to utilize a cost-effective electron source. The most efficient Cr(VI) transforming enzymes that have been described to date are flavoproteins [16]–[20], but these require expensive NAD(P)H cofactors to power reduction, and stoichiometric addition of these cofactors is not an economically viable proposition for large scale applications [21].

To address all of these issues we: (i) screened a previously assembled library of NAD(P)H dependent bacterial oxidoreductase enzymes to identify the most effective Cr(VI) reducing candidate; (ii) showed that a cofactor regenerating enzyme partner can be employed to provide a renewable and inexpensive supply of reduced NAD(P)H to drive Cr(VI) transformation; and (iii) employed a biological system for immobilizing enzymes to polyhydroxyalkanoate (PHA) beads [22] as an economical means of generating large quantities of stable and active Cr(VI) reductase.

## Materials and Methods

### Bacterial strains, growth conditions, plasmids and oxidoreductase genes

*E. coli* strains and plasmids used in this study are listed in Table 1. All *E. coli* strains were grown in Luria broth (LB) supplemented with glucose (1% w/v), ampicillin (50 μg/ml), kanamycin (50 μg/ml) or chloramphenicol (75 μg/ml) as appropriate. For PHA bead production, BL21 cells were initially grown at 37°C in 1 L LB supplemented with glucose (1% w/v), ampicillin (100 μg/ml), and chloramphenicol (75 μg/ml). When the culture reached an OD_600_ of 0.3, fusion protein expression was induced by the addition of 1 mM isopropyl-β-D-thiogalactopyranoside (IPTG). Following induction, cells were transferred to 25°C and cultivated for 44 h to allow production of PHA beads. For oxidoreductase library screening in whole cell lysates, candidate genes were over-expressed from plasmid pUCX in *E. coli* SOS-R1 as described [23]. The full list of candidate genes in the 45-membered oxidoreductase library (ordered alphabetically by bacterial strain each was cloned from) was as follows: *Bacillus subtilis* (ATCC 6051) *nfrA, ycnD, ydgI, yfkO; Enterobacter cloacae* (ATCC 13047) *nfsI; Escherichia coli* (W3110) *azoR, kefF, mdaB, nemA, nfsA, nfsB, wrbA, ycaK, ycdI, ydjA, yieF; Klebsiella pneumoniae* (ATCC 13883) *nfsA, nfsB, nemA, ydjA; Nostoc punctiforme* (PCC 73102) *nfsA, ycdI; Nostoc sp.* (PCC 7120) *ycdI; Pseudomonas aeruginosa* (PAO1) *azoR (PA1962), chrR (PA1204), msuE (PA2357) nfsB (PA5190), ycaK (PA1225); Pseudomonas putida* (KT2440) *azoR (PP4538), kefF (PP3720), nfsA (PP2490), nfsB (PP2432); Pseudomonas syringae pv. phaseolicola* (1448a) *azoR, mdaB, wrbA; Salmonella typhi* (ATCC l9430) *azoR, nfsA, nfsB; Vibrio fischeri* (ATCC 7744) *nfsB (FRase I); Vibrio harveyi* (ATCC 33843) *frp (flavin reductase P); Vibrio harveyi* (KCTC 2720) *frp (flavin reductase P); Vibrio vulnificus* (ATCC 27562) *azoR, nfsA, nfsB, nemA*. To distinguish genes or enzymes with the same name, for the purpose of this work each oxidoreductase was referred to using standard nomenclature followed by an underscore and a two letter abbreviation of the genus and species, e.g. NfsA_Kp and NemA_Ec for NfsA enzyme from *K. pneumoniae* and NemA enzyme from *E. coli*, respectively (exceptions being NfsI_Ecl for NfsI from *E. cloacae*, and Frp_Vh1 and Frp_Vh2 to distinguish the flavin reductase P variants from *V. harveyi* ATCC 33843 and KCTC 2720, respectively).

**Table 1:** Bacterial strains and plasmids used in this study.

Strain or plasmid	Description	Source
*E. coli*		
BL21	F-, *ompT*, *hsdS*_B_ (r_B_- m_B_-), *gal*, *dcm*	Novagen
BL21(DE3)	F-, *ompT*, *hsdS*_B_ (r_B_- m_B_-), *gal*, *dcm* (DE3)	Novagen
SOS-R1	Strain W3110 F^-^ *lac*-6(del)λΦ(*sfiA*::*lacZ*), *nfsA, nfsB*	[23]
Plasmids		
pUCX	pUC19 derivative containing *lacI*, *tac* promoter, *lac* operator, RBS region and *rrnB* terminator from pMMB67EH. Ap^R^.	[23]
pET28a^+^	His6-tag expression vector. Km^R^.	Novagen
pMCS69	pBBR1MCS derivative containing genes *pha*A and *pha*B of *R. eutropha* colinear to *lac* promoter, Cp^R^.	[43]
pET-14b:PhaC	pET-14b derivative containing gene *pha*C of *R. eutropha* colinear to T7 promoter, Ap^R^.	[44]
pET-14b:PhaC-L-NemA_Ec	pET14b derivative containing gene *nem*A of *E. coli* fused to the 3′ end of *pha*C by a linker sequence.	This study

### Protein purification

Recombinant His6-tagged oxidoreductases and GDH from *B. subtilis* (ATCC 6051) were expressed from pET28a^+^ in *E. coli* BL21(DE3) cells and purified by nickel-affinity chromatography employing the His•Bind kit (Novagen, Merck, Darmstadt, Germany) as per the manufacturer's instructions. All enzymes were recovered at >90% purity as judged by SDS-PAGE analysis. For enzyme kinetic analyses, eluted fractions containing visible quantities of enzyme (judged by eye based on the yellow color of the associated flavin cofactor) were pooled and incubated on ice with an excess of pure FMN (FAD for MdaB_Ec) for at least 1 h before buffer-exchange into 40 mM Tris–Cl (pH 7.0) using a 5 ml HiTrap™ desalting column (GE Healthcare Bio-Science, Uppsala, Sweden). Based on initial rate assessment in whole cell lysates the following oxidoreductases were selected and purified in this manner: NfsA_Kp, NfsA_St, YcnD_Bs, Frp_Vh1 (NfsA family); YdgI_Bs, NfsB_Kp, FRaseI_Vf, NfsB_Vv, NfsB_St (NfsB family); NemA_Ec (NemA family); MdaB_Ec (MdaB family); ChrR_Pa, ChrR_St (ChrR/YieF family); KefF_Pa (KefF family); AzoR_Ec (AzoR family). For activity analysis in conjunction with cofactor regenerating partners or with substrates other than Cr(VI), NemA_Ec was purified and treated as described above, but eluted and desalted post FMN treatment into 50 mM sodium phosphate buffer (pH 7.0) rather than Tris-Cl (pH 7.0). The reason for this is that the FDH purchased from Sigma-Aldrich (St Louis, USA) is recommended for use in sodium phosphate buffer; moreover, we empirically determined that GDH likewise rapidly loses activity if stored in 50 mM Tris-Cl (pH 7.0), but not 50 mM sodium phosphate buffer (pH 7.0) (not shown). Like NemA_Ec, His6-tagged GDH was purified using the His•Bind kit as per the manufacturer's instructions, but immediately desalted after elution into 50 mM sodium phosphate buffer (pH 7.0), containing 1 M NaCl (empirically found to enhance stability of purified GDH enzyme; not shown). Protein concentrations were calculated using the DC protein assay kit (Bio-Rad, Hercules, CA) and enzyme purity was confirmed by SDS-PAGE. Purified proteins were stored at 4°C and all reactions were performed within 1–2 weeks of initial purification, to prevent loss of enzyme activity through degradation or precipitation.

### Cr(VI) reductase assays

#### Kinetic assessment of purified Cr(VI) reductase activity

Cr(VI) reductase activity was measured by a modified 1,5-diphenyl carbazide assay [24]. Reactions were performed at 22°C in replicates with a final volume of 0.3 ml containing 1 mM NADH, 50 mM Tris-Cl (pH 7.0) buffer, a range of chromate concentrations (0–200 μM) and sufficient enzyme to transform 100 μM of Cr(VI) within 2–10 min for the 200 μM replicate. The reaction was initiated by addition of 1 mM NADH and the solution was mixed by resuspending with a pipette for 10 s. Immediately after mixing the first 50 µl aliquot was added to a cuvette containing 950 μl of color developing solution (0.1N H_2_SO_4_ and 0.01% 1,5-diphenylcarbazide) and this was taken as t = 0. Subsequent 50 µl aliquots from the reaction were stopped every 30 s thereafter in identical fashion. The absorbance of each sample was then read at 540 nm and the residual Cr(VI) concentration was determined by comparison with a standard curve. The reaction velocity was estimated by the amount of chromate reduced over a two minute period, unless this was found not to be linear in which case the enzyme concentration was adjusted appropriately and the replicate repeated. All reactions were performed in duplicate and kinetic parameters were calculated from a Lineweaver-Burk plot in Microsoft Excel. 1 mM NADH was found to be effectively saturating for each of NemA_Ec, Frp_Vh1, and NfsA_Kp (e.g. *K_m_* of NemA_Ec was measured as 450 µM; Figure S1 in File S1).

#### Measurement of Cr(VI) reduction rate in the presence of crude extract or lawsone

The enzymes AzoR_Ec, NemA_Ec and NfsA_Kp were tested for their ability to reduce Cr(VI) in the presence of cell lysate or lawsone. Cell lysate assays were carried out at 22°C in 100 μl volumes, containing 150 μM Cr(VI), 500 μM NADH, 0.03 mg/ml enzyme in a mixture of either 0∶1, 1∶2, 2∶1 or 1∶0 *E. coli* cell lysate and 50 mM Tris-Cl buffer (pH 7.0). Reactions were stopped at 5 min (NemA_Ec) or 30 min (NfsA_Kp or AzoR_Ec) by addition of 150 µl 2x concentrated color developing solution (i.e. 0.2 N H_2_SO_4_ and 0.02% 1,5-diphenylcarbazide), and residual Cr(VI) concentration determined at 540 nm by comparison to a standard curve. Cr(VI) reduction rates in the presence or absence of lawsone were determined in an identical fashion to the “no cell lysate” replicates above, but with addition of lawsone to a final concentration of 0, 50, 100, 150 or 200 µM.

#### Supply of reduced cofactor via FDH or GDH mediated cofactor regeneration

Non-stoicheometric Cr(VI) reduction by NemA_Ec in the presence of limiting NADH or NAD^+^ was achieved at 22°C in 0.5 ml volumes containing 100 mM sodium phosphate buffer (pH 7.0), 15 µg of purified recombinant NemA_Ec or 65 µg of PHA beads displaying NemA_Ec, 150 μM of K_2_CrO_4,_ 0.05 mM of NADH or NAD^+^, and 15 µg of either FDH +5 mM formic acid or GDH +5 mM glucose. Reduction of chromate was monitored over time as for the purified enzyme kinetic assays above.

#### Synthesis and purification of NemA_Ec fused PHA beads

PHA beads displaying covalently attached NemA_Ec were generated exactly as per the MalE-displaying PHA beads described by Jahns and Rehm [25]. To achieve this, the *malE* gene was excised from plasmid pET-14b PhaC-linker-MalE and replaced by *E. coli nemA*, amplified by PCR using the following primers: *Forward* CCCCCTCGAGATGTCATCTGAAAAACT G; *Reverse* CCCCGGATCCTTACAACGTCGGGTAATC (the underlined sequences indicate the recognition sequences for the *Xho*I and *Bam*HI restriction enzymes used to replace *malE* with *nemA*, yielding plasmid pET-14b PhaC-linker-NemA). During PHA synthesis (growth conditions as described above), the polyhydroxyalkanoate synthase PhaC becomes covalently attached to the surface of the resulting polyester bead, and hence the NemA_Ec fusion partner was also displayed in this manner. The fidelity of the PCR amplification and molecular cloning process was confirmed by DNA sequencing of the inserted *nemA* gene and surrounding plasmid regions (Macrogen, Korea). The successful production of polyester beads was determined by staining cultures with Nile Red and observing cells with fluorescence microscopy as previously described [26]. The *E. coli* cells containing polyester were then mechanically disrupted and the lysate was subjected to centrifugation (4,000×*g*, 20 min, 4°C) to sediment the PHA beads. Final separation of the PHA beads from bacterial lysate was achieved by ultracentrifugation on a glycerol gradient [27]. SDS-PAGE and scanning densitometry indicated that the fusion protein PhaC-NemA_Ec constituted ca. 22% of the total protein present in the bead suspension (with other major species having previously been identified as co-purified host cell proteins; BHA Rehm, unpublished data). NemA_Ec functionality was confirmed by Cr(VI) reductase (1,5-diphenyl carbazide) assay as described above. Isolated PHA beads (wet weight of just over 1.5 g isolated from the 1 L *E. coli* culture) were stored in potassium phosphate buffer (pH 7.5) with 20% ethanol at 4°C until required.

### Assessment of the stability of bead-immobilized NemA_Ec

Purified PHA beads displaying NemA_Ec were tested for their ability to reduce Cr(VI) over time. Reactions were performed at 22°C in replicates with a final volume of 0.3 ml containing 150 µM or 50 µM Cr(VI), 1 mM NADH and 50 mM sodium phosphate buffer (pH 7.0). The reaction was initiated by addition of 65 µg beads and 50 µl aliquots were taken at 30 s intervals and added to 950 μl of color developing solution to measure Cr(VI) concentration, as previously. Reactions were performed in duplicate on a weekly basis for 12 weeks and again after 30 and 36 weeks, and rates of reactions were compared across the entire time period.

### *N*-ethylmaleimide and CB1954 reduction assays

To monitor *N*-ethylmaleimide reduction, reactions were performed in a 1 ml volume containing 200 µM NADPH, 10 µl of either 0.34 µg.ml^−1^ purified NemA_Ec or NemA_Ec PHA bead suspension, and 100 µM *N*-ethylmaleimide. After 10 min incubation at 22°C the concentration of *N*-ethylmaleimide reduced was calculated based on the concentration of NADPH oxidized (assuming a 1∶2 stoichiometry), using an extinction coefficient for NADPH of 6.2×10^3^ M^−1^cm^−1^ at 340 nm. To monitor CB1954 reduction, reactions were performed in a 1 ml volume containing 200 µM NADPH, 10 µl of either 0.34 µg.ml^−1^ purified NemA_Ec or NemA_Ec PHA bead suspension, and 100 µM CB1954. After 10 min incubation at 22°C the concentration of CB1954 reduced was calculated based on equal absorbance of both possible hydroxylamine reduction products of CB1954 at 420 nm; ε  = 1.2×10^3^ M^−1^cm^−1^ [28].

## Results

**Figure 1:**
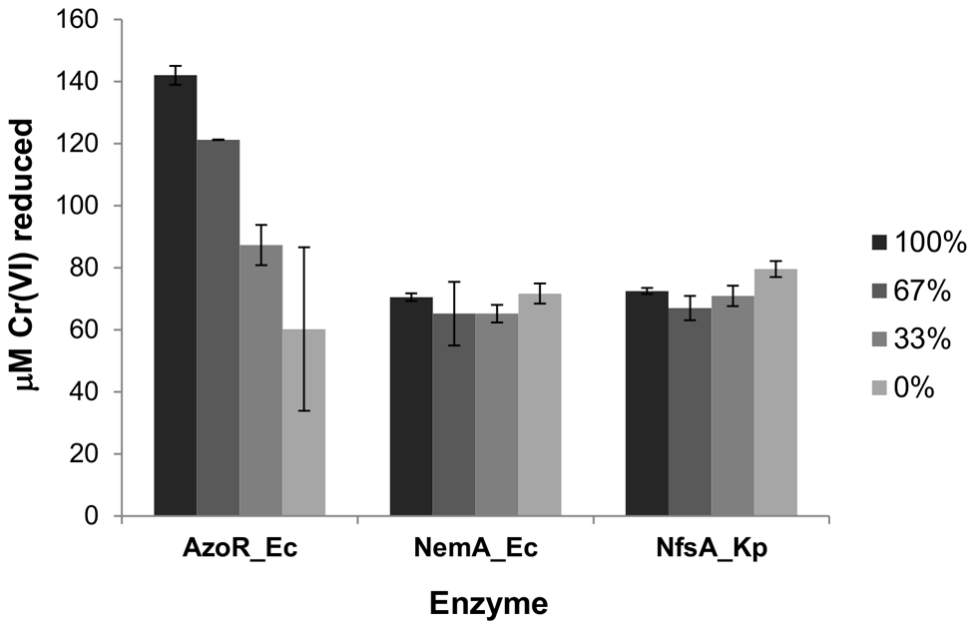
Effect of adding cell lysate on Cr(VI) reductase activity of purified enzymes. Reactions contained 150 μM Cr(VI), 500 µM NADH, and 0.03 mg/ml enzyme in a total volume of 100 μl made up with either 100%, 67%, 33% or 0% *E. coli* cell lysate as indicated in the key (the remaining volume being made up by 50 mM Tris-Cl buffer pH 7.0). Reactions were stopped at 5 min (NemA_Ec) or 30 min (NfsA_Kp and AzoR_Ec) by addition of 1,5-diphenylcarbazide color developing solution, and the amount of Cr(VI) reduced was calculated. Data are the mean of two independent replicates, and error bars indicate ±1 standard deviation.

In ongoing work aiming to discovering effective nitroreductases for cancer gene therapy we have assembled a large collection of bacterial oxidoreductases, over-expressed from plasmid pUCX in *E. coli* W3110 [23], [29]. As previous results have indicated that Cr(VI) reduction is likely a promiscuous activity of flavoenzymes in general [19], [30], we screened a representative sub-library from this collection (45 oxidoreductases, derived from 14 different bacterial species and comprising 12 distinct enzyme families; details in Methods and Materials) to identify the most effective Cr(VI) reducing enzymes. In preliminary screens, however, we observed little to no correlation between the over-expressed Cr(VI) reductase activity measured in *E. coli* cell lysates and the activity of selected enzyme candidates at a purified protein level (not shown). Several of our most active enzymes in cell lysates, in particular those from the AzoR enzyme family, were nearly inactive as purified (His6-tagged) proteins. This was initially surprising to us, as previous directed evolution studies had shown a strong correlation between the activities of clonal variants measured at a cell lysate and purified protein level [31]. However, AzoR from *E. coli* (AzoR_Ec) has previously been described as an azoreductase whose activity is greatly enhanced in the presence of quinone redox mediators such as lawsone [32]. Based on this we hypothesized that quinones or other redox mediators present in *E. coli* cell lysate might be transferring electrons to Cr(VI) via indirect reduction pathways. The activity of purified AzoR_Ec was therefore re-tested in mixtures of 0∶1, 1∶2, 2∶1 and 1∶0 *E. coli* cell lysate and Tris-Cl buffer (pH 7.0), and we found that the velocity of Cr(VI) reduction substantially increased with increasing levels of cell lysate. In contrast, enzymes such as NemA from *E. coli* (NemA_Ec) [23] and NfsA from *Klebsiella pneumoniae* (NfsA_Kp) [33] were unaffected by the addition of cell lysate (Figure 1). Similar results were observed when lawsone was added to enzyme reaction mixtures, with the rate of Cr(VI) reduction by AzoR_Ec substantially increased by lawsone addition, whereas NfsA_Kp was unaffected, and NemA_Ec activity was inhibited by this quinone (not shown).

From these observations we concluded that cell lysate screens were not an appropriate means for comparing the Cr(VI) reducing potential of unrelated enzymes. Instead, we expressed and purified (as His6-tagged recombinant proteins) 15 oxidoreductases we had previously cloned into plasmid pET28a^+^ (derived from seven different flavoenzyme families in total; details in Methods and Materials). Measurement of the Michaelis-Menten kinetic parameters for each purified recombinant enzyme revealed that NemA_Ec was substantially the fastest Cr(VI) reductase, possessing a 4- to 15-fold greater apparent *k_cat_* and greater specificity constant (*k_cat_/K_m_*) for chromate than any of the other enzymes tested (Table 2) (representative Michaelis-Menten and Lineweaver-Burk plots are illustrated in Figure S1 in File S1). In contrast, purified AzoR_Ec in the absence of added cell lysate or lawsone was 2–3 orders of magnitude less efficient. An old yellow enzyme family member from *Thermus scotoductus* SA-01 is the only native soluble oxidoreductase to have previously been measured as having a greater specificity constant for chromate reduction than NemA_Ec [18]. Although NemA_Ec was previously shown to have a preference for NADPH over NADH as cofactor for reduction of several substrates, including *trans*-2-hexenal [34] and the anti-cancer prodrug CB1954 (5-(aziridin-1-yl)-2,4-dinitrobenzamide) [23], no strong cofactor preference was apparent with K_2_CrO_4_ as a substrate (Table 2). Likewise, no substantial cofactor preference was observed for the NfsA-family enzymes Frp_Vh1 and NfsA_Kp (not shown).

**Table 2:** Kinetic parameters for purified recombinant oxidoreductases with Cr(VI) as substrate.

Enzyme^a^	*K_M_* (μM)^b^	*k_cat_* (s^−1^)^b^	*k_cat_/K_M_* (M^−1^s^−1^)
NemA_Ec (NADH)	16±9	1. ±0.1	(1.1±0.6)×10^5^
NemA_Ec (NADPH)	23±4	2.1±0.1	(9.4±1.2) ×10^4^
Frp_Vh1	3.8±0.5	0.32±0.05	(8.4±1.7) ×10^4^
NfsA_Kp	1.7±1.0	0.12±0.03	(6.9±2.7) ×10^4^
NfsB_Kp	47±4	0.37±0.03	(7.8±1.0) ×10^3^
YdgI_Bs	63±1	0.48±0.10	(7.7±1.5) ×10^3^
YcnD_Bs	73±2	0.37±0.03	(5.0±0.5) ×10^3^
FRaseI_Vf	160±3	0.44±0.02	(2.7±0.1) ×10^3^
NfsB_St	320±120	0.54±0.19	(1.7±0.9) ×10^3^
ChrR_Pa	1300±100	1.5±0.1	(1.1±0.1) ×10^3^
MdaB_Ec	170±120	0.20±0.11	(1.1±1.0) ×10^3^
ChrR_St	37±2	0.031±0.001	(8.3±0.4) ×10^2^
KefF_Pa^c^	140	0.06	4.3×10^2^
NfsB_Vv^c^	2200	0.80	3.6×10^2^
NfsA_St^c^	180	0.02	1.4×10^2^
AzoR_Ec^c^	380	0.03	8.7×10^1^

a Enzyme nomenclature is described in full in Materials and Methods. With the exception of NemA_Ec, all enzyme kinetics were measured with NADH as cofactor on the basis that this cofactor is more cost-effective for high level use than NADPH. NemA_Ec kinetic parameters were measured with both cofactors to enable any preference to be identified.

b Apparent *K_M_* and *k_cat_* as measured at 1 mM NADH (or 1 mM NADPH, for NemA_Ec only). Data are the mean of two independent replicates, and error bars indicate ±1 standard deviation.

c No error bars are available, as derivation of meaningful values from a Lineweaver-Burk plot for these low activity enzymes required the replicate data to be averaged prior to analysis.

All purified enzyme assays described in the preceding paragraph were performed at approximately physiological pH, 7.0, in 50 mM Tris-Cl buffer. Serendipitously, this was found to be close to the optimal pH for Cr(VI) reduction by NemA (Figure S2A in File S1). Moreover, there was no significant difference in the velocity of Cr(VI) reduction by NemA_Ec in replicate reactions buffered with either 50 mM Tris-Cl or 50 mM sodium phosphate (p>0.05, T-test) (Figure S2B in File S1). At pH 7.0, no spontaneous reduction of Cr(VI) by direct electron transfer from NADH (i.e. in control reactions lacking enzyme) was measurable within the timeframe of these assays (not shown).

**Figure 2:**
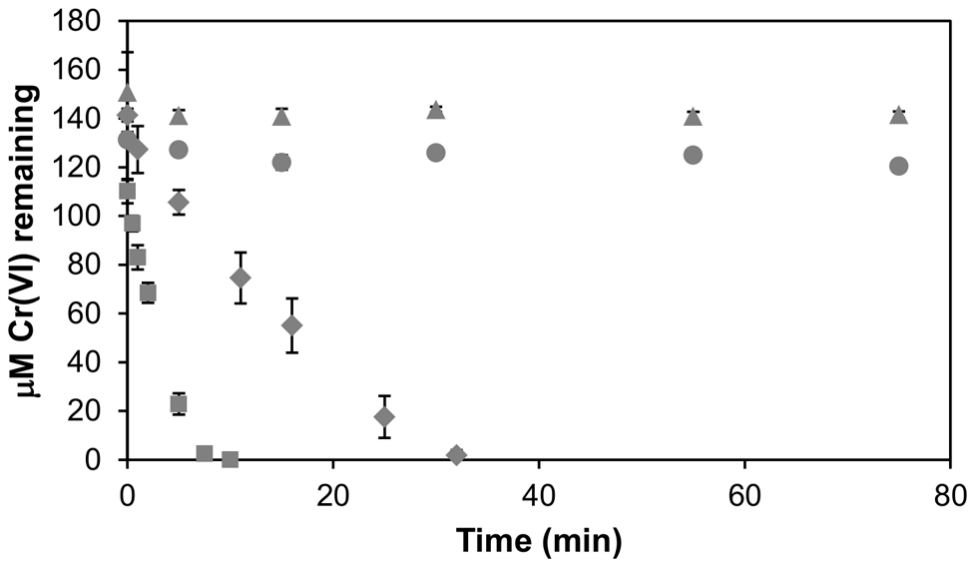
Cr(VI) reduction by NemA_Ec in the presence or absence of a cofactor regenerating partner. 15 µg NemA_Ec were incubated at 22°C with 150 µM potassium chromate in 50 mM sodium phosphate buffer (pH 7.0) and either excess NADH (1 mM; ▪), limiting NADH (50 µM; •), 50 µM NAD^+^ (▴), or 50 µM NAD^+^ together with 15 µg FDH and 5 mM formic acid (♦). Reactions were initiated by addition of NemA_Ec and the concentration of Cr(VI) remaining at each of the time-points indicated was measured by diphenyl carbazide assay. Data are the mean of three independent replicates, and error bars indicate ±1 standard deviation.

Based on these findings, NemA_Ec was taken forward as a preferred Cr(VI) reductase candidate for further study. We first tested whether Cr(VI) reduction by NemA_Ec could be powered by a cofactor-regenerating partner enzyme. As noted previously, the cost of NADH and NADPH cofactors (particularly the latter) is prohibitively expensive for their stoichiometric use in large scale applications. A number of industrial applications that utilize NAD(P)H powered enzymatic transformations therefore employ only small starting quantities of NAD(P)H, and couple the primary reaction to a secondary reaction catalyzed by a partner enzyme such as formate dehydrogenase (FDH) or glucose dehydrogenase (GDH), which continuously regenerate reduced NADH or NAD(P)H, respectively [21]. To test whether FDH and GDH can also be used to power Cr(VI) transformation by NemA_Ec we cloned, expressed and purified GDH from *B. subtilis* as a His6-tagged recombinant protein, and also tested pre-purified *C. boidinii* FDH (Sigma-Aldrich, St Louis, USA). In the absence of a cofactor regenerating partner NemA_Ec: 1) rapidly reduced all of the Cr(VI) in the presence of excess (1 mM) NADH; 2) catalyzed stoichiometric reduction of Cr(VI) in the presence of limiting (50 µM) NADH (ca. 2∶1 molar ratio of NADH oxidized to Cr(VI) reduced); and 3) did not reduce Cr(VI) at all when 50 µM NAD^+^ was added instead of NADH (Figure 2). In contrast, when either FDH and 5 mM formic acid (Figure 2) or GDH and 5 mM glucose (not shown) were added together with 50 µM NAD^+^ then all of the Cr(VI) in the reaction was transformed, demonstrating that both FDH and GDH can be used to regenerate reduced NADH cofactor to power Cr(VI) reduction by NemA_Ec. In control reactions containing only (i) 150 µM K_2_CrO_4_, 50 mM sodium phosphate buffer (pH 7.0), 5 mM formic acid and 1 mM NADH (final pH of mixture  = 6.9); or (ii) 150 µM K_2_CrO_4_, 5 mM formic acid and 1 mM NADH without buffer (final pH of mixture  = 3.0), no spontaneous reduction of Cr(VI) by direct electron transfer from NADH or due to low pH was measurable within the timeframe of these assays (Figure S3 in File S1).

Having demonstrated that cofactor regeneration can be used to supply purified chromate reductase with reduced NADH cofactor, we next sought to develop an economical and scalable method for immobilizing NemA_Ec to a matrix amenable to bioremediation applications. It has previously been demonstrated that PHA polyester beads ‘decorated’ with specific surface proteins can be generated by genetically fusing a protein of interest to a PHA synthase enzyme. During polymer synthesis the PHA synthase, together with its fusion partner, becomes covalently attached and displayed on the surface of the resulting PHA bead [25], [35]. As PHA beads can constitute up to 80% of the dry weight of a bacterial cell [36] and the covalently bound fusion protein accumulates at high copy number per bead [35], [37], this method provides an efficient and economical ‘one pot’ system for preparing a high yield of immobilized and readily purified enzyme.

**Figure 3:**
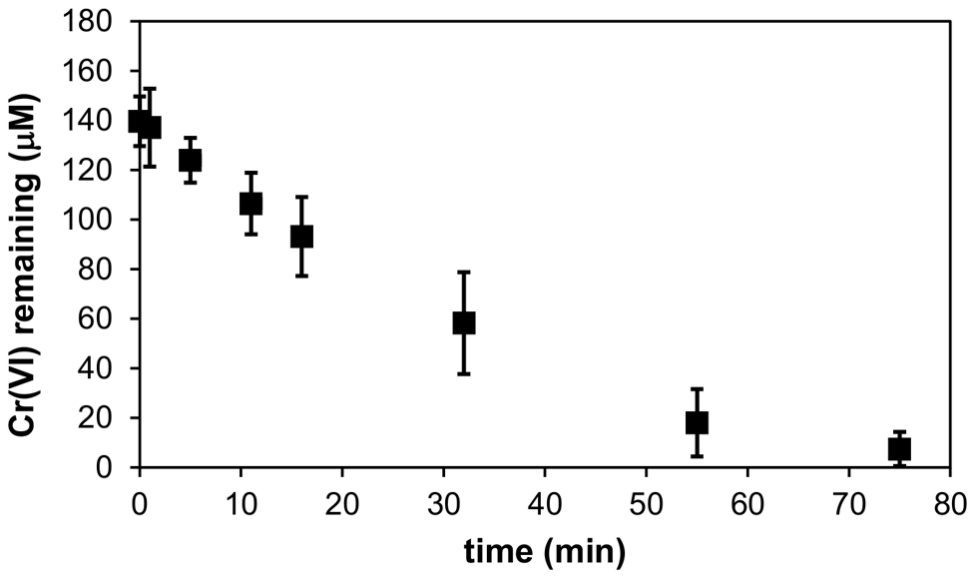
Cr(VI) transformation by PHA beads displaying functional NemA_Ec fused to covalently tethered PhaC from *R. eutropha*. 65 µg of PHA beads displaying NemA_Ec were incubated with 150 µM potassium chromate, 15 µg FDH, 5 mM formic acid and 50 µM NAD^+^ in 0.5 ml of 100 mM sodium phosphate buffer (pH 7) and incubated at 22°C for 75 min. The concentration of Cr(VI) remaining at each of the time-points indicated was measured by diphenyl carbazide assay. Data are the mean of three independent replicates, and error bars indicate ±1 standard deviation.

When *E. coli nemA* was fused to the *phaC* gene from *R. eutropha* and over-expressed together with the *R. eutropha phaAB* genes in *E. coli*, high levels of PHA bead production were observed in cells stained with Nile Red (a hydrophobic fluorogenic dye that associates strongly with PHA; Figure S4 in File S1). To demonstrate that functional NemA_Ec was displayed on the beads, purified beads were incubated with 150 µM Cr(VI), together with FDH, 5 mM formic acid and 50 µM NAD^+^, and complete Cr(VI) transformation was observed (Figure 3). Similar results were observed when purified His6-tagged GDH was employed as a cofactor regenerating partner (not shown). We also sought to generate PHA beads displaying *B. subtilis* GDH, however these beads were not active in glucose metabolism and cofactor regeneration, most likely due to an inability of the PhaC-fused GDH monomers to reconstitute the tetrameric form required for function of this enzyme [38].

**Figure 4:**
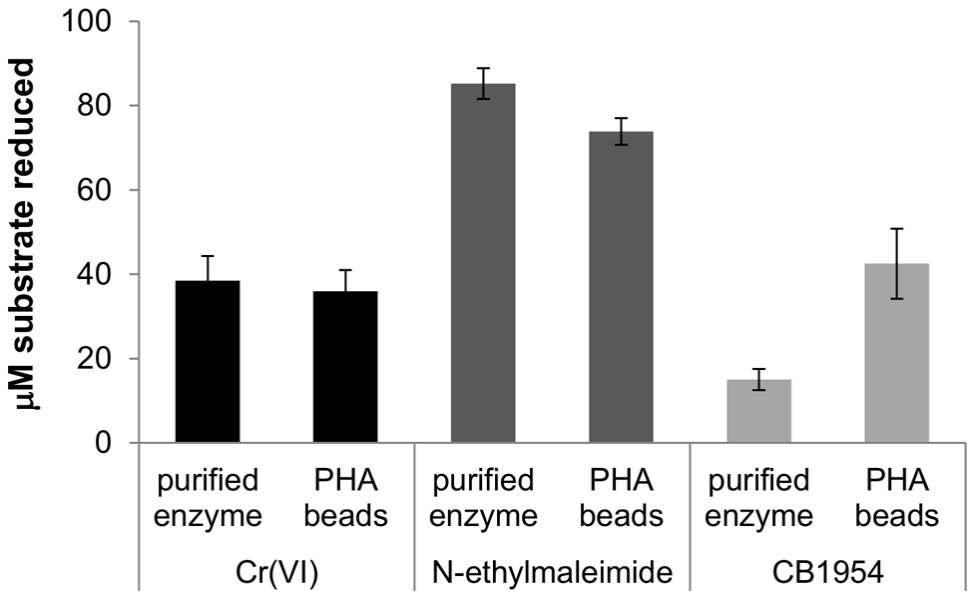
Relative activity of free NemA_Ec *versus* PHA bead immobilized NemA_Ec with different substrates. Replicate reactions were established containing 200 µM NADPH; 10 µl of either 0.34 µg.ml^−1^ purified NemA_Ec or NemA_Ec PHA bead suspension; and 100 µM of either K_2_CrO_4_, *N*-ethylmaleimide, or CB1954. After 10 min incubation at 22°C the concentration of each substrate remaining was measured, enabling the concentration of substrate reduced to be calculated. Data are the mean of three independent replicates, and error bars indicate ±1 standard deviation.

We were unable to estimate the concentration of NemA_Ec protein displayed on the PHA beads, precluding a direct head-to-head comparison of immobilized *versus* free enzyme activities. Instead, we sought to at least determine whether immobilization on PHA beads had compromised NemA_Ec activity. We reasoned that any distortion of the NemA_Ec active site incurred by fusion to PhaC and display on the PHA beads would impact activity with different substrates to a varying extent. To test this we compared the relative activities of free and immobilized enzyme with two other known NemA_Ec substrates (*N*-ethylmaleimide [39] and CB1954 [23]) in addition to Cr(VI). When otherwise identical reactions were established containing either free or immobilized NemA_Ec, and incubated for 10 min with either K_2_CrO_4_, *N*-ethylmaleimide, or CB1954 as substrate, it was found that both free and immobilized enzyme had reduced similar amounts of Cr(VI). In contrast, the immobilized enzyme had reduced substantially more CB1954, but the free enzyme had reduced more *N*-ethylmaleimide (Figure 4). This result suggested that immobilization on the surface of PHA beads had impaired NemA_Ec function to a certain extent, with the degree of impairment less for CB1954 as a substrate than for Cr(VI) or *N*-ethylmaleimide.

Although our inability to accurately measure the concentration of NemA_Ec protein displayed on the PHA beads precluded us from determining the *k_cat_* of the immobilized enzyme, we were still able to measure the *K_m_*. At 1 mM NADH the *K_m_* of immobilized NemA_Ec for Cr(VI) was observed to be 94±26 µM, substantially greater than that of the free enzyme (16±8.6 µM; Table 2). In contrast, the *K_m_* for NADH was effectively unaltered (490±30 µM for immobilized NemA_Ec *versus* 450±30 µM for the free enzyme). This result is consistent with immobilization of Nem_Ec having impaired its ability to transform Cr(VI). Nonetheless, our previous data (e.g. Figure 3) indicate that even immobilized NemA_Ec is still an efficient Cr(VI) reductase. Moreover, with over 1.5 g of beads able to be harvested from just a 1 L culture of *E. coli* grown in LB medium, obtaining sufficient quantities of immobilized NemA_Ec for effective Cr(VI) reduction is unlikely to prove problematic. Finally, we note that the stability of PHA bead immobilized NemA_Ec is high; after 36 weeks of storage at 4°C no discernible reduction in the Cr(VI) reducing activity of the immobilized enzyme was detected (not shown).

## Discussion

While discovery and/or engineering of catalytically superior Cr(VI) reducing enzymes offers scope for improving bacterial bioremediation, there are a number of issues that limit the applicability of such enzymes in a whole-cell context. Not only is Cr(VI) itself toxic to remediating bacteria, it has also previously been shown that expression of enhanced chromate reductase variants in *Pseudomonas putida* or *Escherichia coli* did not significantly improve the rate of chromate transformation by intact cells, whereas it did significantly increase the rate for permeabilized cells and crude cell lysates [31]. This suggests that Cr(VI) uptake by intact cells can become rate limiting, such that improving intracellular Cr(VI) reduction will have no impact on bioremediation potential. Even if these and other problems such as nutrient limitation or the bactericidal effect of other toxins present at polluted sites can be overcome, societal concerns and regulations restricting release of genetically modified organisms will remain a major roadblock.

In contrast an immobilized enzyme-based system for Cr(VI) remediation would not suffer these limitations, but would face additional challenges from the extracellular environment; potential stability issues, a lack of reducing equivalents and – because it presumably cannot self-replicate – a requirement to be able to be produced affordably and in high yield. In this work we addressed each of these issues, identifying an effective and stable Cr(VI) reductase from a bacterial oxidoreductase library; demonstrating a low-cost biological approach for expressing and purifying this enzyme in an immobilized form; and showing that effective Cr(VI) transformation can be powered by only a low starting concentration of cofactor, which is continuously regenerated by a partner enzyme that consumes a cheap sacrificial substrate. We anticipate that the most plausible means of applying this system in Cr(VI) remediation would be for decontamination of groundwater *ex situ*, where administration of enzymes, cofactors and sacrificial substrates could be carefully controlled and the PHA-immobilized enzymes could be easily recovered and re-used. Moreover, as NemA has previously been shown to degrade other environmental pollutants such as the explosives glycerol trinitrate and pentaerythritol tetranitrate [34], the applications of NemA-functionalized PHA beads in environmental remediation could be far more wide-ranging than just Cr(VI) transformation.

Although we were unable to produce functional *B. subtilis* GDH on the surface of PHA beads, most probably because the PhaC-fused monomers were physically constrained from reconstituting the tetramer required for function of this enzyme [38], it is possible that *C. boidinii* FDH, a dimeric enzyme [40] would prove more successful in this regard. A large number of alternative enzyme systems that have been described for regeneration of NAD(P)H cofactors [21] will be considered for future studies. An effective, stable, affordable and easily purified cofactor regenerating enzyme immobilized on PHA beads would doubtless have wide-ranging utility beyond Cr(VI) remediation, with a large number of industrial applications having been described for these enzymes, and immobilization being a prerequisite for most of these [41], [42].

This work also highlighted a disadvantage in using whole cell or crude extract preparations to screen for redox activities catalyzed by highly promiscuous enzymes, in that the desired activity may prove to be indirect, mediated by one or more secondary substrates that may not be relevant to the intended application of the target enzyme. A particular concern for directed evolution studies that seek to improve the activity of such enzymes by an iterative process of mutagenesis and screening is that selected variants may be improved in their metabolism of secondary substrates rather than the primary substrate. That such an outcome was not observed during the previous directed evolution of *E. coli* ChrR to improve chromate reduction [31] suggests that this scenario may not arise if the native enzyme does not already reduce the primary substrate via secondary pathways. As we have demonstrated here, a simple comparison of the activity of the purified native enzyme in the presence or absence of added cell lysate could be used to test for this.

## Supporting Information

File S1 Figure S1. Representative Michaelis-Menten (panels A,C) and Lineweaver-Burk (panels B,D) plots to measure apparent *k_cat_* and apparent *K_m_* for NemA_Ec with Cr(VI) (panels A,B) and NADH (panels C,D) as substrate. Graphs are single repeats of reaction velocity measured at each substrate concentration. The apparent *k_cat_* and *K_m_* with Cr(VI) as substrate were measured at 1 mM NADH; and the apparent *k_cat_* and *K_m_* with NADH as substrate were measured at 150 µM Cr(VI). Figure S2. A. Identification of the pH optimum for NemA_Ec. 15 µg NemA_Ec were incubated at 22°C with 150 µM potassium chromate and 1 mM NADH in either 50 mM sodium phosphate buffer (pH 5.8, 6.5 or 7.0) or 50 mM Tris-Cl (pH 7.5 or 8.8). Reactions were initiated by addition of NemA_Ec and rate of Cr(VI) reduction measured by diphenyl carbazide assay. Data are the mean of three independent replicates, and error bars indicate ±1 standard deviation. B. Comparison of Cr(VI) reduction velocity at pH 7.0 in sodium phosphate buffer or Tris-Cl buffer. Reactions were established, initiated and monitored as described for A, except that either 50 mM sodium phosphate (pH 7.0) or 50 mM Tris-Cl (pH 7.0) were used as buffer. Data are the mean of three independent replicates, and error bars indicate ±1 standard deviation. Figure S3. Control reactions to ensure no spontaneous reduction of Cr(VI) by NADH and/or formic acid. Duplicate reactions of 150 µM K_2_CrO_4_, 5 mM formic acid and 1 mM NADH were incubated with (♦) or without (▪) 50 mM sodium phosphate buffer (pH 7.0). The amount of Cr(VI) remaining in each reaction at each time-point was measured by diphenyl carbazide assay. Data are the mean of three independent replicates, and error bars indicate ±1 standard deviation. Figure S4. Fluorescent micrograph of Nile Red stained *E. coli* producing PHA beads that display NemA_Ec. To visualize PHA beads, 1 ml of a 44 h culture of XLI-Blue cells co-expressing pMCS69 and pET-14b:PhaC-L-NemA_Ec was centrifuged (13, 000 rpm, 1 min) and the pellet resuspended in potassium phosphate buffer (pH 7.5), followed by addition of 10 μl of Nile Red stain (250 μg/ml Nile Red in DMSO). Cells were incubated in the dark for five minutes, pelleted by centrifugation, and re-suspended in potassium phosphate buffer. The micrograph was taken with an Olympus BX51 fluorescence microscope at 1000x magnification using the U-MWIG2 filter set (520–550 nm excitation wavelength and a 565 nm cut-on dichromatic mirror).
(DOCX)

## References

[1] Riley RG, Zachara JM, Wobber FJ (1992) Chemical contaminants on DOE lands and selection of contaminants mixtures for subsurface science research. Report DOE/ER-0547T. U.S. Department of Energy, Washington, D.C.

[2] Okrent D, Xing L (1993) Future risk from a hypothesized RCRA site disposing of carcinogenic metals should a loss of societal memory occur. J Hazard Mater 34: 363–384.

[3] Zhitkovich A (2011) Chromium in drinking water: sources, metabolism, and cancer risks. Chem Res Toxicol 24: 1617–1629. 21766833 10.1021/tx200251t PMC3196244

[4] Richard FC, Bourg ACM (1991) Aqueous geochemistry of chromium: a review. Water Res 25: 807–816.

[5] Lofroth G, Ames BN (1978) Mutagenicity of inorganic compounds in *Salmonella typhimurium*: arsenic, chromium and selenium. Mutat Res 53: 65–66.

[6] Cheng Y, Holman HY, Lin Z (2012) Remediation of chromium and uranium contamination by microbial activity. Elements 8: 107–112.

[7] Ackerley DF, Barak Y, Lynch SV, Curtin J, Matin A (2006) Effect of chromate stress on *Escherichia coli* K-12. J Bacteriol 188: 3371–3381. 16621832 10.1128/JB.188.9.3371-3381.2006 PMC1447458

[8] Brown SD, Thompson MR, Verberkmoes NC, Chourey K, Shah M, et al (2006) Molecular dynamics of the *Shewanella oneidensis* response to chromate stress. Mol Cell Proteomics 5: 1054–1071. 16524964 10.1074/mcp.M500394-MCP200

[9] Liu KJ, Shi X (2001) In vivo reduction of chromium (VI) and its related free radical generation. Mol Cell Biochem 222: 41–47. 11678610

[10] Stearns DM, Wetterhahn KE (1994) Reaction of Cr(VI) with ascorbate produces chromium(V), chromium(IV), and carbon-based radicals. Chem Res Toxicol 7: 219–230. 8199312 10.1021/tx00038a016

[11] Ramírez-Díaz MI, Díaz-Pérez C, Vargas E, Riveros-Rosas H, Campos-Garcia J, et al (2008) Mechanisms of bacterial resistance to chromium compounds. BioMetals 21: 321–332. 17934697 10.1007/s10534-007-9121-8

[12] Yang C, Cheng Y, Ma X, Zhu Y, Holman H-Y, et al (2007) Surface mediated chromate-resistant mechanism of *Enterobacter cloacae* bacteria investigated by atomic force microscopy. Langmuir 23: 4480–4485. 17371056 10.1021/la062925j

[13] Viti C, Giovannetti L (2007) Bioremediation of soils polluted with hexavalent chromium using bacteria: A challenge. In SN Singh, RD Tripathi (eds.), Environmental Bioremediation Technologies, Springer, 57–75.

[14] Brodie EL, Joyner DC, Faybishenko B, Conrad ME, Rios-Velazquez C, et al (2011) Microbial community response to addition of polylactate compounds to stimulate hexavalent chromium reduction in groundwater. Chemosphere 85: 660–665. 21872904 10.1016/j.chemosphere.2011.07.021

[15] McCarty PL (1994) Ground-water treatment for chlorinated solvents. In J. E. Matthews (ed.), Handbook of bioremediation. Lewis Publishers, Ann Arbor, Mich, 87–116.

[16] Gonzalez CF, Ackerley DF, Park CH, Matin A (2003) A soluble flavoprotein contributes to chromate reduction and tolerance by *Pseudomonas putida* . Acta Biotechnol 23: 233–239.

[17] Kwak YH, Lee DS, Kim HB (2003) *Vibrio harveyi* nitroreductase is also a chromate reductase. Appl Environ Microbiol 69: 4390–4395. 12902220 10.1128/AEM.69.8.4390-4395.2003 PMC169119

[18] Opperman DJ, Piater LA, van Heerden E (2008) A novel chromate reductase from *Thermus scotoductus* SA-01 related to old yellow enzyme. J Bacteriol 190: 3076–3082. 18263719 10.1128/JB.01766-07 PMC2293266

[19] Opperman DJ, van Heerden E (2008) A membrane-associated protein with Cr(VI)-reducing activity from *Thermus scotoductus* SA-01. FEMS Microbiol Lett 280: 210–218. 18218019 10.1111/j.1574-6968.2007.01063.x

[20] Sedlacek V, Kucera I (2010) Chromate reductase activity of the *Paracoccus denitrificans* ferric reductase B (FerB) protein and its physiological relevance. Arch Microbiol 192: 919–926. 20821194 10.1007/s00203-010-0622-4

[21] van der Donk WA, Zhao H (2003) Recent developments in pyridine nucleotide regeneration. Curr Opin Biotechnol 14: 421–426. 12943852 10.1016/s0958-1669(03)00094-6

[22] Rehm BH (2010) Bacterial polymers: biosynthesis, modifications and applications. Nat Rev Microbiol 8: 578–592. 20581859 10.1038/nrmicro2354

[23] Prosser GA, Copp JN, Syddall SP, Williams EM, Smaill JB, et al (2010) Discovery and evaluation of *Escherichia* coli nitroreductases that activate the anti-cancer prodrug CB1954. Biochem Pharmacol 79: 678–687. 19852945 10.1016/j.bcp.2009.10.008

[24] Greenberg AE, Connors JJ, Jenkins D, Franson MA (ed.) (1981) Standard methods for the examination of water and wastewater, 15th ed., p.187–190. American Public Health Association, Washington, D.C.

[25] Jahns AC, Rehm BH (2009) Tolerance of the *Ralstonia eutropha* class I polyhydroxyalkanoate synthase for translational fusions to its C terminus reveals a new mode of functional display. Appl Environ Microbiol 75: 5461–5466. 19581473 10.1128/AEM.01072-09 PMC2737925

[26] Peters V, Rehm BH (2005) In vivo monitoring of PHA granule formation using GFP-labeled PHA synthases. FEMS Microbiol Lett 248: 93–100. 15963662 10.1016/j.femsle.2005.05.027

[27] Jahns AC, Haverkamp RG, Rehm BH (2008) Multifunctional inorganic-binding beads self-assembled inside engineered bacteria. Bioconjug Chem 19: 2072–2080. 18778091 10.1021/bc8001979

[28] Race PR, Lovering AL, White SA, Grove JI, Searle PF, et al (2007) Kinetic and structural characterisation of *Escherichia coli* nitroreductase mutants showing improved efficacy for the prodrug substrate CB1954. J Mol Biol 368: 481–492. 17350040 10.1016/j.jmb.2007.02.012

[29] Prosser GA, Copp JN, Mowday AM, Guise CP, Syddall SP, et al., (2013) Creation and screening of a multi-family bacterial oxidoreductase library to discover novel nitroreductases that efficiently activate the bioreductive prodrugs CB1954 and PR-104A, Biochem Pharmacol doi:10.1016/j.bcp.2013.01.029. 10.1016/j.bcp.2013.01.029 23399641

[30] Ackerley DF, Gonzalez CF, Park CH, Blake R 2nd, Keyhan M, et al (2004) Chromate-reducing properties of soluble flavoproteins from *Pseudomonas putida* and *Escherichia coli* . Appl Environ Microbiol 70: 873–882. 14766567 10.1128/AEM.70.2.873-882.2004 PMC348923

[31] Barak Y, Ackerley DF, Dodge CJ, Banwari L, Alex C, et al (2006) Analysis of novel soluble chromate and uranyl reductases and generation of an improved enzyme by directed evolution. Appl Environ Microbiol 72: 7074–7082. 17088379 10.1128/AEM.01334-06 PMC1636143

[32] Liu G, Zhou J, Wang J, Zhou M, Lu H, et al (2009) Acceleration of azo dye decolorization by using quinone reductase activity of azoreductase and quinone redox mediator. Biores Technol 100: 2791–2795. 10.1016/j.biortech.2008.12.040 19208470

[33] Prosser GA, Patterson AV, Ackerley DF (2010) *uvrB* gene deletion enhances SOS chromotest sensitivity for nitroreductases that preferentially generate the 4-hydroxylamine metabolite of the anti-cancer prodrug CB1954. J Biotechnol 150: 190–194. 20727918 10.1016/j.jbiotec.2010.08.007

[34] Williams RE, Rathbone DA, Scrutton NS, Bruce NC (2004) Biotransformation of explosives by the old yellow enzyme family of flavoproteins. Appl Environ Microbiol 70: 3566–3574. 15184158 10.1128/AEM.70.6.3566-3574.2004 PMC427764

[35] Peters V, Rehm BH (2006) In vivo enzyme immobilization by use of engineered polyhydroxyalkanoate synthase. Appl Environ Microbiol 72: 1777–1783. 16517622 10.1128/AEM.72.3.1777-1783.2006 PMC1393242

[36] Lee SY (1996) High cell-density culture of *Escherichia coli* . Trends Biotechnol 14: 98–105. 8867291 10.1016/0167-7799(96)80930-9

[37] Blatchford PA, Scott C, French N, Rehm BH (2012) Immobilization of organophosphohydrolase OpdA from *Agrobacterium* radiobacter by overproduction at the surface of polyester inclusions inside engineered *Escherichia coli.* . Biotechnol Bioeng 109: 1101–1108. 22170266 10.1002/bit.24402

[38] Weckbecker A, Hummel W (2005) Glucose dehydrogenase for the regeneration of NADPH and NADH. Methods Biotechnol 17: 225–238.

[39] Miura K, Tomioka Y, Suzuki H, Yonezawa M, Hishinuma T, et al (1997) Molecular cloning of the *nemA* gene encoding *N*-ethylmaleimide reductase from *Escherichia coli.* . Biol Pharm Bull 20: 110–112. 9013822 10.1248/bpb.20.110

[40] Schirwitz K, Schmidt A, Lamzin VS (2007) High-resolution structures of formate dehydrogenase from *Candida boidinii.* . Protein Sci 16: 1146–1156. 17525463 10.1110/ps.062741707 PMC2206666

[41] Liu W, Wang P (2007) Cofactor regeneration for sustainable enzymatic biosynthesis. Biotechnol Adv 25: 369–384. 17459647 10.1016/j.biotechadv.2007.03.002

[42] Schmid A, Dordick JS, Hauer B, Kiener A, Wubbolts M, et al (2001) Industrial biocatalysis today and tomorrow. Nature 409: 258–268. 11196655 10.1038/35051736

[43] Amara AA, Rehm BH (2003) Replacement of the catalytic nucleophile cysteine-296 by serine in class II polyhydroxyalkanoate synthase from *Pseudomonas aeruginosa*-mediated synthesis of a new polyester: identification of catalytic residues. Biochem J 374: 413–421. 12924980 10.1042/BJ20030431 PMC1223625

[44] Yuan W, Jia Y, Tian J, Snell KD, Müh U, et al (2001) Class I and III polyhydroxyalkanoate synthases from Ralstonia eutropha and Allochromatium vinosum: characterization and substrate specificity studies. Arch Biochem Biophys 394: 87–98. 11566031 10.1006/abbi.2001.2522

