# Supplementary figures and images for: Escherichia coli NemA Is an Efficient Chromate Reductase That Can Be Biologically Immobilized to Provide a Cell Free System for Remediation of Hexavalent Chromium
Source: PLoS One. 2013 Mar 13;8(3):e59200. doi: 10.1371/journal.pone.0059200 (PMC3596305; doi:10.1371/journal.pone.0059200)

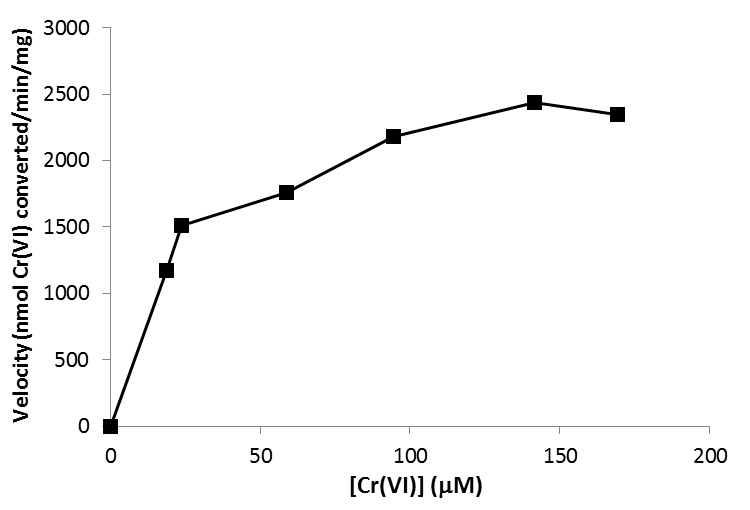
**
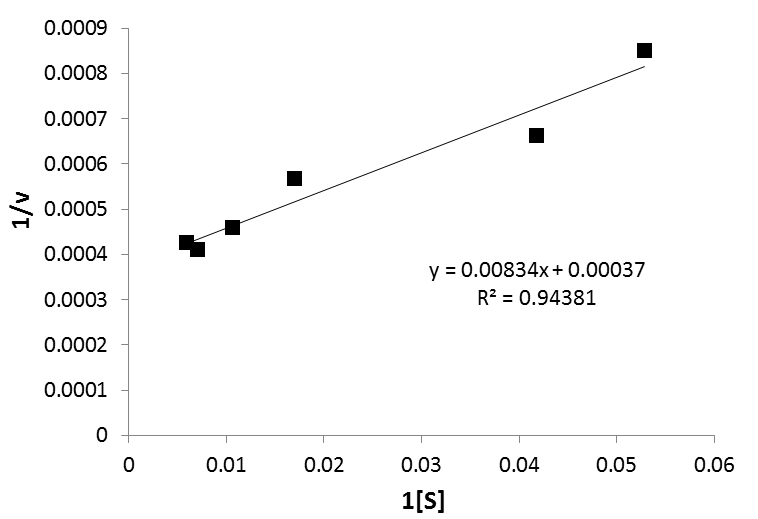
 FIGURE S1**

**
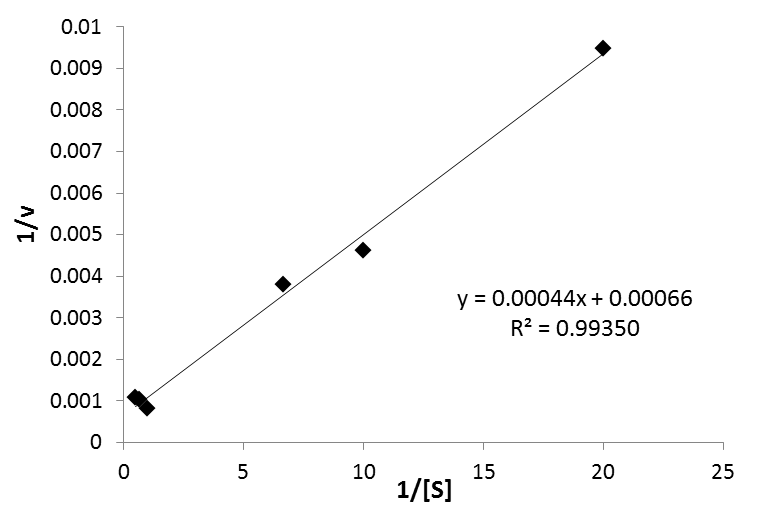
**

**B**

**A**

**D**

**C**

**
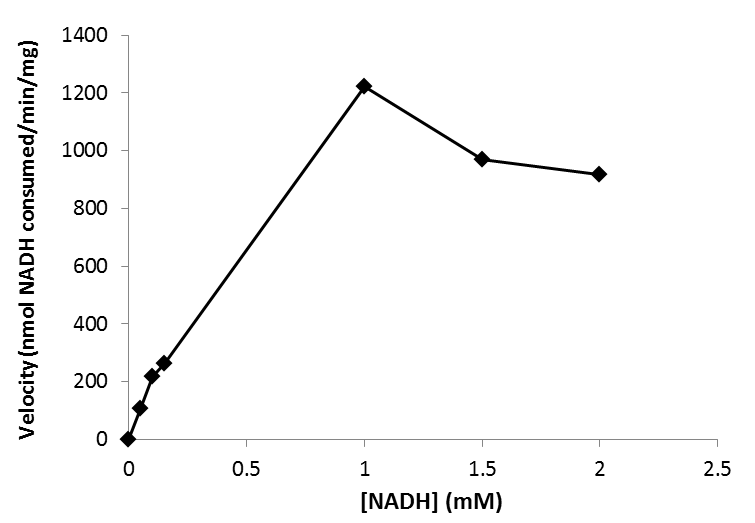
**

**FIGURE S2**

**
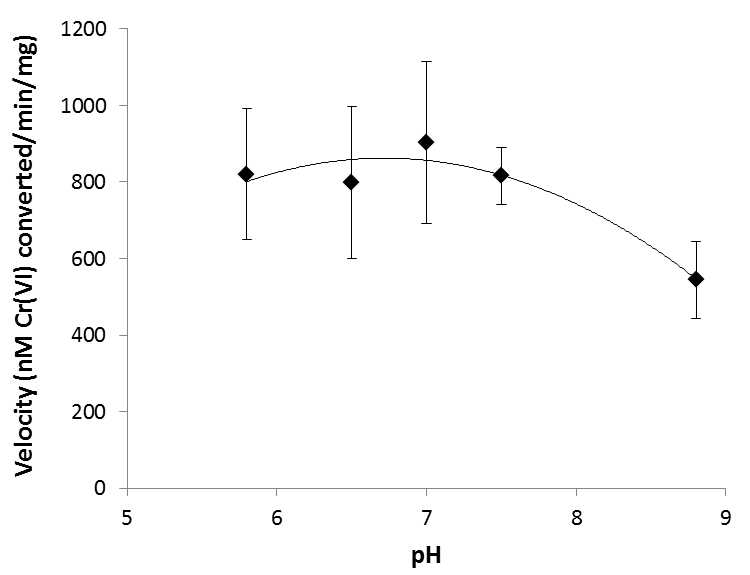

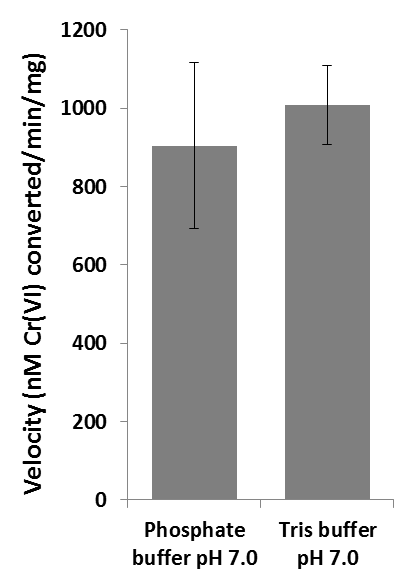
**

**B**

**A**

**FIGURE S3**

**FIGURE S4**

**
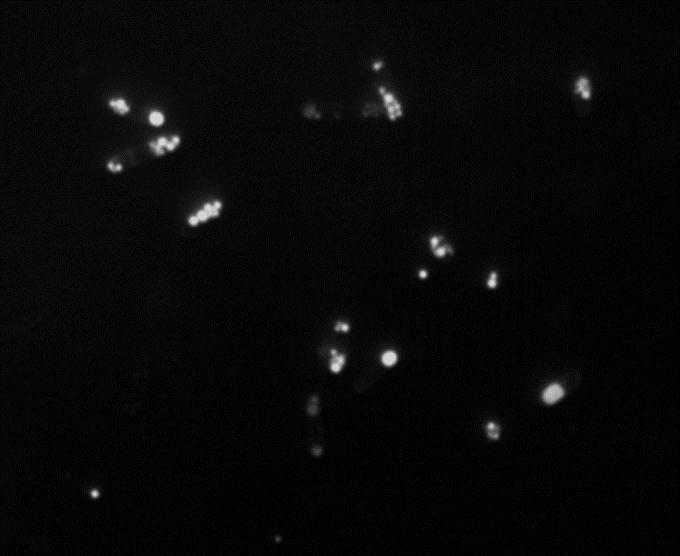
**

Supplement: File S1 — Figure S1. Representative Michaelis-Menten (panels A,C) and Lineweaver-Burk (panels B,D) plots to measure apparent kcat and apparent Km for NemA_Ec with Cr(VI) (panels A,B) and NADH (panels C,D) as substrate. Graphs are single repeats of reaction velocity measured at each substrate concentration. The apparent kcat and Km with Cr(VI) as substrate were measured at 1 mM NADH; and the apparent kcat and Km with NADH as substrate were measured at 150 µM Cr(VI). Figure S2. A. Identification of the pH optimum for NemA_Ec. 15 µg NemA_Ec were incubated at 22°C with 150 µM potassium chromate and 1 mM NADH in either 50 mM sodium phosphate buffer (pH 5.8, 6.5 or 7.0) or 50 mM Tris-Cl (pH 7.5 or 8.8). Reactions were initiated by addition of NemA_Ec and rate of Cr(VI) reduction measured by diphenyl carbazide assay. Data are the mean of three independent replicates, and error bars indicate ±1 standard deviation. B. Comparison of Cr(VI) reduction velocity at pH 7.0 in sodium phosphate buffer or Tris-Cl buffer. Reactions were established, initiated and monitored as described for A, except that either 50 mM sodium phosphate (pH 7.0) or 50 mM Tris-Cl (pH 7.0) were used as buffer. Data are the mean of three independent replicates, and error bars indicate ±1 standard deviation. Figure S3. Control reactions to ensure no spontaneous reduction of Cr(VI) by NADH and/or formic acid. Duplicate reactions of 150 µM K2CrO4, 5 mM formic acid and 1 mM NADH were incubated with (♦) or without (▪) 50 mM sodium phosphate buffer (pH 7.0). The amount of Cr(VI) remaining in each reaction at each time-point was measured by diphenyl carbazide assay. Data are the mean of three independent replicates, and error bars indicate ±1 standard deviation. Figure S4. Fluorescent micrograph of Nile Red stained E. coli producing PHA beads that display NemA_Ec. To visualize PHA beads, 1 ml of a 44 h culture of XLI-Blue cells co-expressing pMCS69 and pET-14b:PhaC-L-NemA_Ec was centrifuged (13, 000 rpm, 1 min) and the pell [file pone.0059200.s001.docx]
